# FGF23 in Chronic Kidney Disease: Bridging the Heart and Anemia

**DOI:** 10.3390/cells12040609

**Published:** 2023-02-13

**Authors:** Andreja Figurek, Merita Rroji, Goce Spasovski

**Affiliations:** 1 Institute of Anatomy, University of Zurich, 8057 Zurich, Switzerland; 2 Department of Nephrology, Faculty of Medicine, University of Medicine Tirana, 1001 Tirana, Albania; 3 University Clinic for Nephrology, Medical Faculty, University St. Cyril and Methodius, 1000 Skopje, North Macedonia

**Keywords:** fibroblast growth factor 23, chronic kidney disease, anemia, iron deficiency, erythropoietin, heart, cardiovascular risk

## Abstract

Fibroblast growth factor 23 (FGF23) is a phosphaturic hormone produced mainly in osteocytes. In chronic kidney disease (CKD) FGF23 levels increase due to higher production, but also as the result of impaired cleavage and reduced excretion from the body. FGF23 has a significant role in disturbed bone and mineral metabolism in CKD, which leads to a higher cardiovascular risk and mortality in these patients. Current research has emphasized the expression of FGF23 in cardiac myocytes, fibroblasts, and endothelial cells, and in addition to the effects on the kidney, its primary role is in cardiac remodeling in CKD patients. Recent discoveries found a significant link between increased FGF23 levels and anemia development in CKD. This review describes the FGF23 role in cardiac hypertrophy and anemia in the setting of CKD and discusses the best therapeutical approach for lowering FGF23 levels.

## 1. FGF23 Signaling and Pathophysiology in CKD

The protein Fibroblast Growth Factor 23 (FGF23), a member of the Fibroblast Growth Factor (FGF) family, controls the metabolism of vitamin D and phosphate in the blood. Another protein that serves as a coreceptor of FGF receptors (FGFRs) is klotho, which is predominantly expressed in the kidney [1]. FGFR1c appears to be the primary receptor for FGF23. The biologically active form of FGF23 consists of two parts: an N-terminal FGF core homology domain (155 amino acids) that shares homology with other members of the FGF family and a C-terminal domain (72 amino acids) that is important for relations with the FGFR-Klotho complex [2]. In addition to the mediation of the binding of FGF23 to de novo sites generated at the complex FGFR1c-Klotho interface, the 72-residue C-terminal tail of FGF23 inhibits full-length ligand binding to binary FGFRs Klotho complex actively suppressing FGF23 activity. This canonical FGF23/FGFR/α-Klotho codependent signaling limits the effects of FGF23 on α-Klotho-expressing tissues [3]. Although FGFR is abundantly expressed throughout the body, Klotho’s tissue expression is tightly regulated (membrane-bound Klotho is expressed predominantly in the distal segment of renal tubules, parathyroid, choroid plexus, the sinus node, and minimally in bone and cartilage), thus, determining the effects of the FGF23 on target organs [4]. Under normal physiological conditions, the kidney maintains soluble Klotho homeostasis, whereas chronic kidney disease (CKD) patients had lower *klotho* mRNA expression in the kidney following the decline of kidney function [5].

FGF23 is secreted primarily by osteocytes to maintain phosphate and mineral homeostasis. Its physiological effect on renal functions is mediated through binding to FGF receptor (FGFR) 1 in the proximal tubular cells in the presence of its co-factor klotho. The complex induces intracellular activation of mitogen-activated protein kinase (MAPK) signaling that leads to augmented renal phosphate excretion and declined 1,25-dihydroxy vitamin D synthesis. Thus, it stimulates phosphate excretion, inhibits parathyroid hormone (PTH) secretion, and lowers active vitamin D levels [6,7]. These adaptations help maintain normal phosphate, but as CKD progresses to end-stage kidney disease (ESKD), they may also exert maladaptive effects associated with the high level of FGF23. The early onset of Klotho deficiency in CKD contributes to renal FGF23 resistance and a maladaptive increase in circulating FGF23. In pathological conditions, FGF23 is found to be abnormally elevated in bone, the heart, the liver, and the kidneys.

## 2. FGF23 and Heart Involvement

According to the K/DOQI CKD categorization, cardiovascular involvement is crucial, starts in the early stages of CKD, and affects around 80% of patients receiving regular hemodialysis [8].

The complex combination of traditional, non-traditional, and emerging CKD-linked risk factors, such as uremic toxins, CKD-mineral and bone disorder (MBD), anemia, hypervolemia, oxidative stress, inflammation, insulin resistance, etc., is reflected throughout the association between cardiovascular disease (CVD) and renal disease [9,10]. Here, the cardiac remodeling during the progression of CKD was termed uremic cardiomyopathy. Volume and pressure overload, as well as the uremic condition itself, which includes left ventricular hypertrophy (LVH), diffuse interstitial fibrosis, and microvascular disease, are the causes of uremic cardiomyopathy in individuals with CKD or ESKD [9,11,12]. 

LVH is the main factor that connects FGF23 with cardiovascular problems in CKD. Faul et al. [13] were the ones who initially identified the detailed mechanism of FGF23-induced LVH which is beyond the canonic way and independent of the klotho mechanism [14]. Indeed, FGF23 may stimulate the hypertrophic development of cardiomyocytes through an action that requires the presence of FGFR4 but not FGFR-1/Klotho [15]. Of note, the high FGF23 levels cause an increase in FGFR4 expression, showing that FGFR4 is responsible for the crucial role of FGF23 in cardiac hypertrophy [15]. As a result of this interaction, FGFR4 is autophosphorylated, and phospholipase C gamma (PLCy) is then activated by binding to the phosphorylated FGFR4 region [16]. Activated PLCy causes calcineurin activation and nuclear factor of activated T-cells (NFAT) dephosphorylation, which is then translocated to the nucleus, following transcription of pro-hypertrophic genes [brain natriuretic peptide (*BNP*), regulator of calcineurin 1 (*Rcan1*), β-myosin heavy chain (*β-MHC*), atrial natriuretic peptide (*ANP*), etc.] [17].

Additionally, the development of cardiac hypertrophy is exacerbated by Klotho deficiency, which is present in CKD [18]. Animal experiments revealed that blocking FGFR4 prevents myocardial hypertrophy even in the presence of a high-phosphate diet [19]. Moreover, ventricular hypertrophy has been demonstrated to be reversible in experimental circumstances [19]. The relevance of FGF23/FGFR4/calcineurin/NFAT signaling in the myocardium was further supported by experimental research in rats that revealed a negative correlation between phosphorylated NFAT and cardiomyocyte size, cardiac FGF23 production, and FGFR4 expression [20].

Further research demonstrated the significance of dysregulated calcium (Ca^2+^) homeostasis and the interaction with FGF23 signaling in ventricular hypertrophy. In ventricular myocytes, acute FGF23 therapy raised intracellular Ca^2+^ by encouraging Ca^2+^ entry through L-type Ca^2+^ channels. Consequently, when force and rate of force creation increase, the cardiac contractility changes, suggesting that long-term disturbance of Ca^2+^ homeostasis in cardiomyocytes may result in contractile dysfunction, heart remodeling, and ultimately, cardiac hypertrophy [21,22]. This pathway was explained in detail in our previous review [22].

FGF23 considerably increases LVH in patients with CKD in a paracrine manner [20]. Myocardium from CKD patients and CKD rats can release FGF23 and have a paracrine impact, indicating that FGF23 may be subject to a local regulating mechanism. In addition, some data show that the paracrine function of FGF23 in cardiac tissue should not be neglected, demonstrating that FGF23 promotes cardiac hypertrophy from numerous angles [R] [23,24,25].

Although FGF23 itself did not directly affect the expression levels of fibrosis-associated mRNAs, it was shown that through inhibited FGFR1 it promotes fibroblast activation TGF-β1, converting fibroblasts into myofibroblasts, a pro-fibrotic crosstalk between cardiac myocytes and fibroblasts [25]. TGF-β is a well-known cytokine that has been shown to cause fibrosis in various tissues by participating in several signaling pathways. According to Smith et al., local renal tubules may produce FGF23, stimulating the injured fibroblast [26]. FGF23 also promotes TGF-β autoinduction and NFAT activation in injury-primed renal fibroblasts, which are components of TGF signaling pathways [27]. These findings point to a paracrine mechanism whereby renal fibroblasts are stimulated to activate pro-fibrotic signaling pathways by FGF23 in the kidney. 

While TGF-β/TGF-β receptor signaling remains constant, FGF23 promotes profibrotic and pro-hypertrophic growth factors in the neonatal rat ventricular myocytes (NRVM) inducing angiotensinogen expression in cardiac myocytes [28]. Overall, these findings imply that FGF23, secreted by cardiac myocytes, may specifically target cardiac fibroblasts, and stimulate profibrotic pathways in a paracrine fashion. Animal models have identified FGF23 as a paracrine factor secreted by cardiomyocytes to promote cardiac fibrosis under conditions where TGF-β is activated [25].

Andrukhova et al. showed that irrespective of changes in serum klotho or serum PTH, circulating intact FGF23 were found elevated, and serum levels of vitamin D hormone were hardly reduced after induction of experimental myocardial infarction (MI) in rat and mouse models. After the MI, FGF23 expression increased in both the skeletal and cardiac tissues. Despite the fact that the molecular link between the cardiac lesion and circulating FGF23 concentrations has not yet been fully explored, the study has discovered a remarkable heart-bone-kidney axis that may have significant therapeutic implications and may establish a new area of cardio-osteology.

It is essential to emphasize that urine excretion of phosphate, calcium, and sodium, as well as blood levels of PTH, phosphate, and sodium, remain unchanged despite the induction of MI and increased FGF23, together with biomarkers such as urine excretion of collagen cross-link deoxypyridinoline, serum alkaline phosphatase, Dickkopf-1, and osteoprotegerin [29]. 

Moreover, in vitro studies in post-MI-rodents, reported that increased cardiac FGF23 expression drives cardiac fibrosis via activation of β-catenin which is a well-known pro-fibrotic factor that interacts with TGF- β signaling [30]. The study by Piersma et al. provides a nice summary of the detailed crosstalk between TGF, and Wnt/β-catenin, but it is beyond the focus of this review [31].

Inflammation may result from a disrupted FGF23/Klotho axis [32], suggesting another way for an indirect FGF23 and Klotho influence on the heart. Experimental models have shown that FGF23 influences the immune system and induces the hepatic production of inflammatory cytokines [33]. FGF23 is thought to impair the host’s response to infection and the innate immune response [34,35]. Independently from klotho, the increased FGF23 effect on FGFR-2 on neutrophils inactivates b2-integrin and prevents neutrophil recruitment to infection sites, which may help to partially explain the compromised host defense brought on by neutrophil dysfunction in CKD patients. Studies have also shown that FGF23 inhibits 1,25-dihydroxy vitamin D3 synthesis, causes macrophage activation, and malfunctions in monocytes (which increases susceptibility to bacterial infection); all events are strongly associated with chronic inflammation [36].

As a result, a relationship between the local and systemic variables controlling FGF23 was observed, leading to the hypothesis that a positive feedback loop may occur in which FGF23 stimulates the release of pro-inflammatory cytokines, that in turn increases the levels of FGF23 secretion. On the other hand, some findings support the idea that inflammation is also a potent inducer of the synthesis of FGF23 and that the cleavage of FGF23 by furin/furin-like proteases is essential for maintaining appropriate iFGF23 levels in response to a concurrent rise in circulating cFGF23 [37].

Initial reports on FGF23 and the heart suggests that FGF23 can indirectly affect cardiovascular (CV) homeostasis through its classical renal activities by the bone- renal-cardiac axis generated by FGF23 “on-target” activation of FGFR/-Klotho complexes in the kidney, since -Klotho is abundantly expressed in kidneys but not in the heart. The activation of renin-angiotensin-aldosterone system (RAAS) was indicated to have direct hypertensive effects and promote pro-hypertrophic, pro-fibrotic, and pro-inflammatory ways in cardiac cells [38]. Aldosterone, angiotensin II (Ang II), and beta-adrenergic pathways make up the afferent limb of this bone-renal-cardiac axis, stimulating FGF23 gene expression in bone. It is interesting to note that administering Ang II to rodents increased the ectopic expression of FGF23 in the heart, a tissue that typically does not express FGF23 or Klotho in physiological amounts [39,40], as well as the expression of the FGF23 in bone, the physiological site of FGF23 production. Additionally, the injection of Ang II raises the amount of FGF23 in the blood [41]. High levels of FGF23 were reported in patients with heart failure who were not receiving angiotensin-converting enzyme inhibitors (ACEi) medications. Furthermore, patients in the third tercile of elevated serum FGF23 showed a lower risk of adverse events after treatment with ACEi, underlining the clinical importance of crosstalk between RAAS and FGF23 [42]. Therefore, FGF23 might directly activate RAAS by inhibiting ACE2 [43], and research suggests that RAAS activation can also boost FGF23 production [44]. Thus, through the pathological signaling processes involving cardiac myocytes and immune cells, angiotensin II, and aldosterone, as active RAAS components, FGF23 may induce vascular and myocardial fibrosis and cardiac hypertrophy apart from the effect on elevated blood pressure [25].

Moreover, the increased FGF23 may be caused by direct effects that activate angiotensin 1 (AT1) receptors in osteoblasts or secondary effects that stimulate the production of aldosterone and tumor necrosis factor (TNF) as a result of Ang II, both of which can increase FGF23 or suppress the expression of Klotho in the kidney, leading to an end-organ resistance of FGF23 and progressively FGF23 elevation [41,45]. In addition, the sympathetic nervous system ‘SNS’ stimulates the production of FGF23 in bone via the β-adrenergic signaling pathway [45]. 

Additionally, the efferent limb consists of FGF23 activation of renal tubular FGFRs/α-Klotho by at least four mechanisms such as induction of hypertension through activation of RAAS; suppression of 1,25(OH)_2_ vit D, which together with tissue vitamin D receptor R(VDR) expression as a result of FGF23’s inhibition of 1-α hydroxylase activity promoting renin overexpression with the activation of the ACE/Ang II arm of the RAAS; ectopic expression of FGF23 and αKlotho in the stressed myocardium; finally, through the loss of cardioprotective effects of sKL in LVH [22,46] all with well-known effects in the myocardium. In this context, a recent thorough review highlighted the interactions between mineralocorticoid receptor (MR) activation and changes in the FGF23/Klotho axis, revealing the mechanisms through which both excess FGF23 and MR activation may induce cardiorenal damage. Mineralocorticoid receptor antagonists (MRAs) may work to counteract and quiet these linked mediators, which in turn would help to increase the cardiorenoprotective efficacy of both existing and newly developed therapeutic paradigms [47]. The increased levels of both local and circulating FGF23 may lead to endothelial dysfunction, small vessels, and further influence the development of CVD in CKD [48].

Furthermore, another link was discussed between renal function, vascular calcification, and FGF23 expression in heart disease patients [49] The presence of vascular FGF23, FGFR1, FGFR3, and α-klotho in both the tunica intima and media characterizes calcification areas co-localized interestingly, with inflammatory macrophages [49]. 

Moreover, FGF23 stimulates ADAM17 expression, which leads to the release of soluble s-klotho by the cleavage of membrane-bound α-klotho. As part of the research into the endothelial cell function targeted by FGF23, it was demonstrated that in the presence of α-klotho, the AKT/endothelial NO synthase (eNOS) pathway is activated in vitro by FGF23 to cause the production of nitric oxide (NO). Additionally, the stimulation of the NADPH oxidase 2 (Nox2)/p67phox/p47phox/Rac1 signaling complex by FGF23 therapy increases the production of reactive oxygen species (ROS) [50]. 

These findings imply that the effects of FGF23 on endothelial function are FGFR-dependent in the presence of α-klotho. Inhibition of α-klotho using a neutralizing antibody might specifically block FGF23-mediated activation of AKT/eNOS and, consequently, the release of NO. It suggests that in states of Klotho deficiencies, such as CKD, FGF23-induced NO synthesis might be blunted, and ROS formation might overrule ROS degradation.

Recent research has emphasized how FGF23 is expressed in cardiac myocytes, cardiac fibroblasts, and endothelial cells, and how this may affect normal and abnormal heart development and cardiac function. Furthermore, they discovered for the first time that high blood pressure causes cell type-specific regulation of FGF23 in heart tissue, revealing increased production in cardiac fibroblasts and endothelial cells. Further research should explore the precise molecular processes of FGF23, the interaction between cardiac and non-cardiac myocytes, and the influence of these factors on cardiac physiology and disease in both in vivo and in vitro contexts [23]. Additionally, compared to therapy with active vitamin D, the study of Dorr et al. demonstrated that etelcalcetide inhibits LVH development in hemodialysis patients with secondary hyperparathyroidism. The observations are consistent with the idea that FGF23, apart from its effects on the kidney, is the primary direct cause of cardiac remodeling in CKD patients. The results of this study may have a significant impact on patients and policymakers due to the marked global illness burden of people with LVH and CKD [51]. 

**Figure 1:**
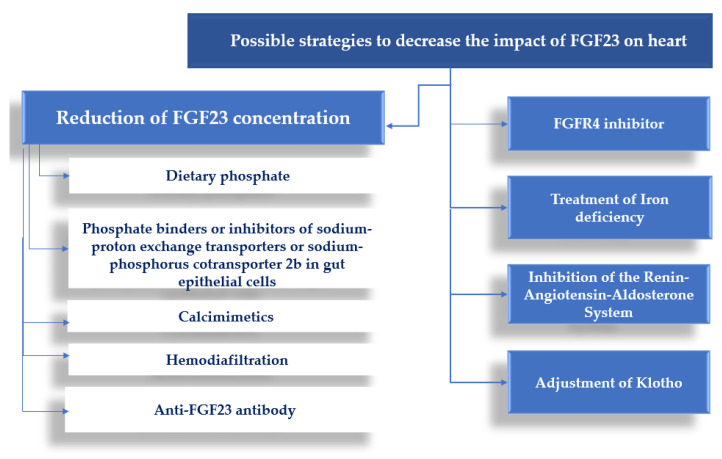
The potential approach for reducing the FGF23 effect on the heart.

Furthermore, all the potential strategies to decrease phosphate and subsequently the level of FGF23 should be considered [52,53,54]. Taken together, the possibilities for reducing the effect of FGF23 on the heart are depicted in Figure 1.

## 3. FGF23 and Anemia in Patients with CKD

Anemia is a frequent complication in patients with CKD and is a result of both decreased erythropoietin (EPO) production by kidney peritubular fibroblasts and iron deficiency. Iron deficiency in CKD can be either true or functional. True iron deficiency occurs in the setting of malnutrition, impaired dietary absorption by medications, blood losses from phlebotomy, hemodialysis, and uremic platelets, whereas functional iron deficiency is caused by inflammation-induced hepcidin, the iron regulatory hormone that causes degradation of ferroportin (the iron exporter) in order to block iron release from iron recycling hepatic and splenic macrophages, hepatic iron stores, and dietary sources into the bloodstream [55]. Recent research revealed an important bidirectional crosstalk between FGF23, known as a major regulator of phosphate homeostasis, with iron deficiency and EPO production. 

The first link between iron deficiency and FGF23 was found in patients with autosomal dominant hypophosphatemic rickets, where FGF23 mutations that impair cleavage occur, resulting in hypophosphatemia [56]. Thereafter, it has been demonstrated that iron chelators stimulate FGF23 production in isolated bone cell cultures with the transcriptional regulation by hypoxia-inducible factor 1α (HIF 1α) as playing a mechanistical role [57]. In addition to iron deficiency and inflammation, EPO is shown to increase FGF23 production. After injecting human recombinant EPO, circulating total FGF23 increased in humans and rodents, but was not followed by the increase of intact FGF23 (iFGF23) [58], since EPO stimulates both FGF23 production and cleavage. However, in a CKD mouse model where FGF23 cleavage is reduced, EPO injection caused an increase in the iFGF23 with the reduced percentage of iFGF23 relative to total FGF23, indicating that EPO stimulates FGF23 cleavage in CKD [59].

FGF23 also regulates iron homeostasis and erythropoiesis, having both direct and indirect actions. Experimental data showed that FGF23 directly reduces the EPO secretion from kidneys which results in a decreased differentiation of erythroid progenitors (pro-erythroblasts) to mature erythrocytes [60]. Moreover, FGF23 enhances erythrocyte apoptosis by reducing the fraction of erythrocytes in the G2/M phase of the cell cycle [60]. FGF23 deletion in mice increased renal EPO expression, circulating EPO levels, hemoglobin, and red blood cell number, as well as bone marrow erythroid progenitors and hematopoietic stem cells [61]. Indirectly, increased FGF23 stimulates the production and secretion of pro-inflammatory cytokines (IL-6, IL-1β), which in turn upregulate hepcidin production in the liver [60]. In a CKD mouse model, FGF23 signaling inhibition improved anemia and iron deficiency by increasing EPO levels, hemoglobin, red blood cells, bone marrow erythroid progenitors, and driving hematopoietic stem cells to the erythroid lineage [62].

**Figure 2:**
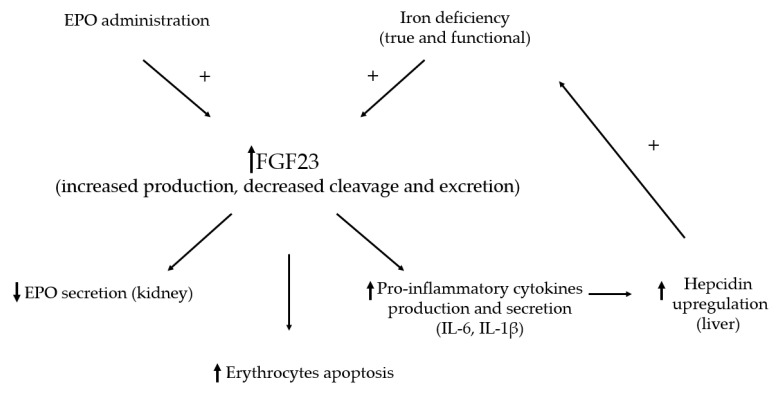
FGF23 and anemia in chronic kidney disease. Abbreviations: EPO—erythropoietin, IL-6—interleukin 6, IL-1β—interleukin 1 beta. The symbol “+” indicate an enhancing effect, arrows ↑ and ↓ indicate an increase and decrease respectively.

The crosslink between FGF23 and anemia is summarized in Figure 2.

Recent findings from the rodent experiments and clinical trials showed that treating anemia may be beneficial in reducing FGF23 levels. Experimental data in CKD rats showed that iron-based phosphate binder ferric citrate lowered plasma phosphate, iFGF23, and indicated a trend toward lower cFGF23 [63]. Ferric citrate treatment in CKD mice improved cardiac function by reducing FGF23 levels [64]. Moreover, not only ferric citrate but also intravenously applied iron sucrose, reduced aorta and heart calcifications [63]. However, both iron and phosphate correction as well as FGF23 levels reduction contributes to better outcomes by improvement in anemia and vascular calcifications, respectively. 

Clinical data in non-dialysis CKD patients indicated the association between total FGF23 levels with the risk of incident and prevalent anemia, and a decline in hemoglobin over time [65,66]. A randomized control trial on the safety and efficacy of ferric carboxymaltose versus ferumoxytol to treat iron deficiency showed that both of these intravenous iron preparations in the subgroup of patients with CKD reduced total FGF23 levels accompanied by improvements in hemoglobin and iron parameters. Ferric carboxymaltose also increased iFGF23 levels that were related to renal phosphate wasting, increased PTH, hypophosphatemia, and reduced serum calcium and calcitriol [67]. However, in patients with CKD, the risk of hypophosphatemia is reduced, as they have a lower ability to excrete phosphate [67]. When compared to ferric gluconate, with ferric carboxymaltose treatment reduced EPO doses were administered achieving stable and target values of hemoglobin [68]. It should be underlined that ferric carboxymaltose has a more stable carbohydrate shell, only partially broken after the administration with a gradual and more stable release of iron into the blood, avoiding precocious transferrin saturation [68]. Moreover, ferric carboxymaltose treatment does not cause complement activation during hemodialysis, indicating its low pro-inflammatory activities [68,69].

In non-dialysis CKD patients iron-based phosphate binder ferric citrate improved hemoglobin and iron parameters with a decrease in serum phosphate and both iFGF23 and total FGF23 [70]. Post hoc analysis in this study revealed that reduction in iFGF23 was partially mediated by a decreased serum phosphate, and total FGF23 reduction was partially mediated by increased transferrin saturation [71]. In hemodialysis patients, ferric citrate significantly reduced iFGF23 levels compared to lanthanum carbonate, although serum phosphate, calcium, and PTH levels were unchanged in both groups, suggesting that the decrease in iFGF23 levels were caused by improvement in iron deficiency [72]. The EVOLVE trial demonstrated that a 30% reduction in iFGF23 in patients with CKD was associated with a reduction in all-cause mortality and adverse vascular outcomes [73].

In the setting with normal kidney function, iron deficiency, and EPO increase the production and cleavage of FGF23. On the other hand, CKD is a known condition of increased FGF23 production and reduced degradation. Moreover, the present iron deficiency (either true or functional) with anemia or EPO administration, contributes to high iFGF23 levels, that influence phosphate, PTH, and vitamin D metabolism defined as chronic kidney disease-mineral and bone disorder (CKD-MBD). Iron supplementation can, thus, influence the FGF23 levels. In contrast to oral iron therapy, intravenous iron forms increase iFGF23 levels by decreasing its cleavage [74]. However, both oral and intravenous iron therapy decrease cFGF23 levels. Thus, a clinical study of 225 pre-dialysis CKD patients that were not on erythropoiesis-stimulating agents or intravenous iron therapy showed an inverse association between cFGF23 and hemoglobin levels [75].

In addition to iron supplementation, treatments with the aim to increase EPO levels and, thus, correct anemia in CKD and the association with FGF23 have been investigated. Recent preclinical and clinical data highlighted a new approach to treating patients with CKD anemia. Compared to the erythropoietin stimulating agents (ESA), which use is well established in the clinical practice, but with a risk of major adverse events (cardiovascular, cerebrovascular, thrombosis), hypoxia-inducible factor prolyl hydroxylase (HIF-PH) inhibitors are under clinical investigation. HIF is a major regulator of EPO production in a response to hypoxia and its activity is limited by the prolyl hydroxylase enzyme. It has been shown that HIF-PH inhibitors cause more physiological increase in EPO levels compared to ESA, increases serum hemoglobin, decreases hepcidin and ferritin levels, decrease transferrin saturation by increasing total iron-binding capacity, and have a lower risk of major adverse events [76]. Experimental data showed that treatment with EPO and HIF-PH inhibitors in anemic mice with CKD reduced iFGF23 levels by more than 70% without causing hyperphosphatemia, improved iron utilization, and restored the balance of genes responsible for 1,25 vitamin D production [77]. Increased EPO, either exogenous or endogenous, stimulates erythroid precursors to secrete erythroferrone (ERFE), which decreases hepcidin concentration by acting directly on the liver and improves iron utilization [78]. 

High cardiovascular risk in CKD, mediated by increased FGF23 levels, includes two mechanisms: pro-hypertrophic effects and systolic dysfunction occurrence and progression. Whereas pro-hypertrophic effects are a result of FGFR4/PLCγ/calcineurin/NFAT signaling activation, the onset of systolic dysfunction is mediated by increased FGFR3 expression and MAPK signaling activation [15,64].

Since FGF23 increases cardiovascular risk and mortality in CKD, anti-FGF23 antibodies were tested in experiments in rats with CKD to reduce FGF23 levels. These antibodies lead to an increase in proximal tubular phosphate reabsorption and a subsequent increase in serum phosphate [79]. However, hyperphosphatemia resulted in developing ectopic calcification and higher mortality [80]. Therefore, the use of anti-FGF23 antibodies in the setting of CKD-MBD could be deleterious for CKD patients. 

## 4. Conclusions

**Figure 3:**
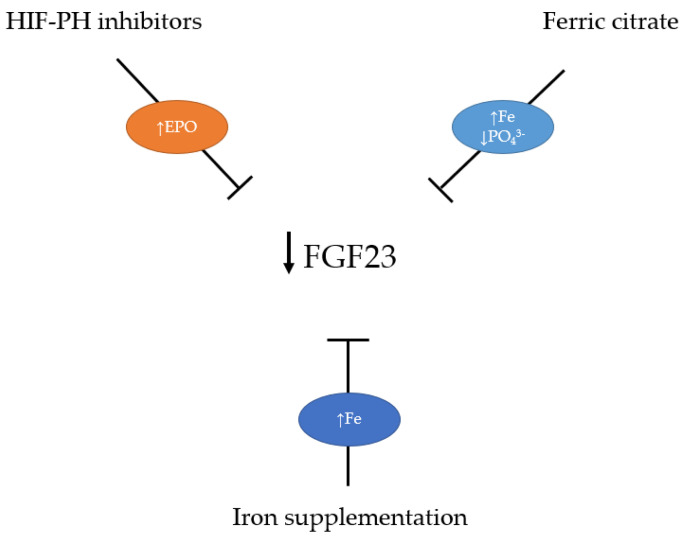
Therapeutic approach for FGF23 lowering in chronic kidney disease. Abbreviations: EPO—erythropoietin, HIF-PH—hypoxia-inducible factor prolyl hydroxylase, Fe—iron, PO_4_^3−^—phosphate.

Taken together, the increased FGF23 levels in early CKD stages might have beneficial effects by reducing serum phosphate and cardiovascular risk. When CKD progresses, increased FGF23 signaling contributes to increased cardiovascular risk in these patients, and reduction of FGF23 levels is desirable. Recent research revealed that iron metabolism plays a crucial role in modulating pathophysiological FGF23 signaling. Therefore, it seems that probably an indirect lowering of FGF23 levels through correction of iron deficiency, anemia, and hyperphosphatemia by iron-based phosphate binders and induction of more physiological EPO production might have a better effect than direct blocking of FGF23 signaling (Figure 3). 

## Data Availability

Not applicable.
